# An analysis of outcome measures in a specialist inpatient eating disorders unit in Aberdeen: changes since 2015 and response to the COVID-19 lockdown

**DOI:** 10.1192/bjo.2021.491

**Published:** 2021-06-18

**Authors:** Emma Cross, Jane Morris

**Affiliations:** 1 University of Aberdeen; 2 Royal Cornhill Hospital

## Abstract

**Aims:**

Identify differences in outcome measures between inpatient cohorts during the first year of the pandemic compared with preceding years.

Identify key elements in the treatment provided by identifying any trends in the outcome measures between 2015 and 2021.

**Background:**

The Eden Unit at the Royal Cornhill Hospital (RCH) in Aberdeen is a ten-bed specialist centre for the inpatient treatment of eating disorders (ED). Strict measures to control the spread of COVID-19 have meant that important aspects of therapy in the Eden Unit are no longer permissible. It is not known whether handicaps to providing the previous service are reflected in recent outcomes.

**Method:**

Values for age, length of stay (LOS), BMI, HbA1c (diabetic patients) and responses to three questionnaires: Eating Disorder Evaluation Questionnaire (EDE-Q); Depression Anxiety Stress Scale (DASS)-21; and Clinical Outcomes in Routine Evaluation (CORE). This data were collected for April 2020 to February 2021 (Pandemic) and compared with five preceding years, April 2015 to March 2020 (Pre-COVID). The project was registered with NHS Grampian Quality Assurance Team and approved by the MCN Quality Assurance subgroup. Ethical approval was not required. A data collection sheet allowed anonymised data to be entered into a Microsoft Excel TM Spreadsheet for analysis of baseline demographics.

**Result:**

Average age of patients remained similar across the six years. Length of stay in the first year of the pandemic was significantly shortened. BMI on discharge in 2020/21 remained similar to preceding years. If relevant, HbA1c was measured throughout admission and comparison with BMI change reflected a focus on treating both diabetes and ED concurrently. Comparison of admission and discharge questionnaires to determine outcome measures proved difficult due to the small number of responses to both.

**Conclusion:**

Shorter LOS during the pandemic was a significant finding. Despite this, BMI on discharge remained similar, suggesting a shift to weight restoration due to lack of opportunities for an holistic approach due to restrictions. Key elements of treatment include careful monitoring of HbA1c and concurrent management of Type I Diabetes for those patients. The low response rate to questionnaires raises concern regarding their use, in their current format, as effective tools to measure outcomes. Though low numbers of questionnaire responses prevent firm conclusion, it appears that the reduced opportunities for elements of treatment to be undertaken in the community may have contributed to increased anxiety levels on discharge.

